# Observations on Luminous Animals

**Published:** 1811-06

**Authors:** J. Macartney


### *Observations on Luminous Animals.* By J. MACARTNEY, Esq.

(Concluded from page 424.)

THE phenomenon of animal light has been attempted to be explained in different ways. By many persons it was formerly ascribed to a putrefactive process, but since the modern theories of combustion became known, it has been generally believed to depend upon an actual inflammation of the luminous substance, similar to the slow combustion of phosphorus. Others have accounted for the luminous effect, by supposing the matter of light to be accumulated, and rendered latent under particular circumstances, and afterwards evolved in a sensible form.

The opinion of the light of living animals being the consequence of putrefaction, is evidently absurd, and contradictory to all observation on the subject. It has been proved by the experiments of Dr. Hulme and others, that even the luminous appearances of dead animals are exhibited only during the first stages of the dissolution of the body, and that no light is emitted after putrefaction has really commenced.

Spallanzani, who was the most strenuous advocate for the phosphorescent phosphorescent nature of animal light, stated that glow-worms shone more brilliantly when put into oxygen gas; that their light gradually disappeared in hydrogen or in azotic gas, and was instantly extinguished in fixed air; that it was also lost by cold, and revived by the application of a warm temperature. He conjectured that the luminous matter of these insects was composed of hydrogen and carbonated hydrogen gas.

Forster relates, in the Lichtenberg Magazine for 1783, that on putting a lampyris splendidula into oxygen gas, it gave as much light as four of the same species in common air.

Carradori has made some experiments upon the lucciole, (lampyris italica) which led him to deny its phosphorescence. He found that the luminous portion of the belly of the insect shone in vacuum, in oil, in water, and different liquids, and under different circumstances, where it was excluded from all the communication with oxygen gas. He accounts for the result of Forster's experiment, by supposing, that the worm shone more vividly, because it was more animated in oxygen gas than in common air.

Carradori adopts on this subject the doctrine of Brugnatelli, and ascribes the luminous appearances of animals, to the condensation and extrication of light in particular organs, which had previously existed in combination with the substance of their bodies. He supposes the light to be originally derived from the food, or the atmospheric air taken into the body; in short, that certain animals have the peculiar property of gradually imbibing light from foreign bodies, and of afterwards secreting it in a sensible form.*

The following experiments which I made upon this subject, would lead me to make different conclusions than those of the preceding authors.

*Experiment* 1.—A glow worm was put into a glass of water, in which it lived nearly two hours, and continued to emit light as usual, until it died, when the luminous appearance entirely ceased.

*Experiment* 2.—The luminous substance was extracted from the beforementioned glow-worm, and from others killed in different ways, but it afforded no light.

*Experiment* 3.—The sacs containing the luminous matter were cut from the bellies of *living* glow-worms, and shone uninterruptedly for several hours in the atmosphere, and after their light became extinct, it was revived by being moistened

* Annal di Chimica, Tomo xiii. 1797.

with with water; some of these were put into water in the first instance, in which they continued to shine unremittingly for 48 hours.

*Experiment* 4.—The luminous substance of a glow-worm was exposed to a degree of heat which would have been sufficient to inflame phosphorus, without increasing the brilliancy of its light; and farther, it could not be made to burn by being applied to a red hot iron, or to the flame of a candle.

*Experiment* 5.—A delicate thermometer was introduced amongst some living glow-worms, during the time they gave out much light: the temperature of the room being 69, the instrument rose to 75, 76, and 77, according to circumstances, as the warmth was reflected from the hand, or dissipated by the worm crawling over cold substances. The luminous portion of the tail, when very brilliant, appeared to raise the thermometer more quickly than the other parts of the body, but it was not invariably the case. When shining strongly, I thought that the luminous rings communicated the sensation of warmth to the hand, but this was probably a deception, as the actual degree of heat was not sufficient for such an effect. It should however be mentioned, that in Templar's observations on the glow-worm, he said his feelings deceived him, if he did not experience some heat from the shining of the insect.*

*Experiment* 6.—To satisfy myself how far the evolution of heat during the shining of glow-worms, depended upon the life of the animals, I cut off the luminous portion of the tail from several living worms, and I found that if the thermometer was applied to them immediately, it was raised by them one or two degrees; but after these parts were dead, although they continued to emit light, they produced no effect whatever upon the instrument.

*Experiment* 7.—Some hemispherical medusæ were put into a spoon, containing a small quantity of sea water, and held over a burning candle. As soon as the water became heated the medusæ appeared like illuminated wheels, the spots at the margin and center alone emitting light; in which manner they shone vividly and permanently for about 20 seconds, when they shrunk and died, after which they were no longer luminous.

*Experiment* 8.—Some of the same species were put into spirits: a strong unremitting light was instantly given out, which issued from the central and marginal parts, as in the preceding experiment, and continued until they died.

* Phil. Trans. No. 75.

*Experiment*

*Experiment* 9.—Some of the scintillating and hemispherical species of medusa, contained in a small glass jar, were introduced into the receiver of an air pump, and the air being exhausted, they shone as usual when shaken ; if any difference could be perceived, the light was more easily excited, and continued longer in vacuum.

I wished next to try the influence of electricity on the luminous property of animals.

*Experiment* 10.—A medusa hemispherica was placed in a small glass dish, containing a quantity of water, merely sufficient to allow the animal to preserve its figure; being insulated, it was electrified, and sparks drawn from it, which had not the slightest effect; the experiment was repeated several times with different individuals, but without exciting the animals to throw out light.

*Experiment* 11.—Some hemispherical medusæ were placed in contact with the two ends of an interrupted chain, and slight electric shocks passed through them. During the very moment of their receiving the shock no light was visible, but immediately afterwards the medusæ shone like illuminated wheels, which appearance remained for some seconds. Upon the closest inspection with a magnifying glass, no contractile motion could be perceived to accompany the exhibition of the light. The application of electricity in this instance seems to have acted merely as a strong mechanic shock.

The above experiments on the luminous medusæ were made at Herne, with the assistance of George May, Esq. of Stroud-house, and in the presence of a large company, capable of accurately distinguishing their results.

It seems proved by the foregoing experiments, that so far from the luminous substance being of a phosphorescent nature, it sometimes shews the strongest and most constant light, when excluded from oxygene gas; that it in no circumstances undergoes any process like combustion, but is actually incapable of being inflamed ; that the increase of heat, during the shining of glow-worms, is an accompaniment, and not an effect of the phenomenon, and depends upon the excited state of the insect; and lastly, that heat and electricity increase the exhibition of light, merely by operating like other stimuli upon the vital properties of the animal.

In confirmation of these opinions, I may quote the high authority of the Secretary of this Society, who has found that the light of the glow-worm is not rendered more brilliant in oxygene, or in oxygenated muriatic gas, than in common air; and that it is not sensibly diminished in hydrogene gas.

I may further add, that Spallanzani's experiments of diffusing the luminous liquor of the medusa in water, milk and other fluids, are in direct contradiction of his own theory, as is also the extinction of the light of these mixtures by the application of a high degree of heat.

If the light emitted by animals were derived from their food, or the air they respire, as supposed by CARRADORI, the phenomenon should be increased or diminished, according to the quantity of food or air, that the creatures consume; but we do not find this to be the case; for in those situations where they are sometimes found to be most luminous, they are deprived, in a great measure, of these assumed sources of their light.

In fact, the luminous exhibitions of living animals are not only independent of all foreign light, but are frequently destroyed by the latter. I have always found the shining of the medusæ to cease upon the rising of the moon, or at the approach of day; and when out of the sea, I never could excite them to throw out light until they had been kept for some time in the dark; all the luminous insects likewise secrete themselves as much as possible during the day time, and go abroad only at night. I have, it is true, found that the scolopendra electrica will not shine unless it has been previously exposed to solar light; but I have observed that it shone as brilliantly and as frequently, after being kept a short time in a light situation, as when left unrecovered the whole day. The circumstance of the scolopendra requiring exposure previous to its giving out light, is very unaccountable, as the insect, when left to itself, always seeks as much as possible concealment during the day; indeed it is the opinion of some naturalists that it is killed by the light of the sun.

The opinions of Brugnatelli and Carradori are connected with some general doctrines, respecting the nature of light, which I shall not at present venture to discuss. It appears to me, that the question is still unresolved, whether light has a substantial existence, or is a phenomenon depending upon certain operations or conditions of the ordinary forms of matter. But the highly ingenious researches of Count Rumford, on the laws of what had been called subtile fluids, and the extraordinary advances lately made by Mr. Davy, on the decomposition of substances, that were hitherto looked upon as elementary, give us reason to hope, that future investigations may unfold views of the material world, of which we can at present have only an indistinct conception; that new modes of analysis may enable us to see things, not "through a glass darkly," but more nearly as they are; and that the boundaries of physical and metaphysical science, now so far asunder, may be made to approach each other.

In the present state of our knowledge, our business should be be, to collect, arrange, and compare phenomena, rather than to speculate upon their nature. Nevertheless, I cannot refrain from observing, that the circumstances attending the luminous appearance of living animals, are much more favourable to the supposition of light being a property, than a substance. The quantity of light emitted by an animal in a certain time, (admitting it to be matter) far exceeds that which could be possibly supplied by the sources, from whence it is usually supposed to be derived. Thus the luminous appearance of some medusæ may be continued with the intermission of short intervals for an indefinite time, notwithstanding the creature be kept in darkness, and without any other food than what a small quantity of filtered sea water would afford. The uninterrupted and long continued light that is sometimes evolved by the luminous sacs, and the ova of the glow-worm, is also inconsistent with the notion of an accumulation and subsequent dispersion of a material substance.

I shall terminate this paper by an enumeration of the several conclusions, that are the result of the observations I have been able to make upon the phenomena of animal light.

The property of emitting light is confined to animals of the simplest organization, the greater number of which are inhabitants of the sea.—The luminous property is not constant, but in general, exists only at certain periods, and in particular states of the animal's body.—The power of shewing light resides in a peculiar substance or fluid, which is sometimes situated in a particular organ, and at others diffused throughout the animal's body.—The light is differently regulated, when the luminous matter exists in the living body, and when it is abstracted from it. In the first case, it is intermitting, or alternated with periods of darkness; is commonly produced or increased by a muscular effort; and is sometimes absolutely dependant upon the will of the animal. In the second case, the luminous appearance is usually permanent until it becomes extinct, after which it may be restored directly by friction, concussion, and the application of warmth; which last causes, operate on the luminous matter (while in the living body,) only indirectly, by exciting the animal.—The luminous matter, in all situations, so far from possessing phosphoric properties, is combustible, and loses the quality of emitting light, by being dried, or much heated.—The exhibition of light, however long it may be continued, causes no diminution of the bulk of the luminous matter. It does not require the presence of pure air, and is not extinguished by other gasses.

The luminous appearance of living animals is not exhausted hausted by long continuance, or frequent repetitions, nor accumulated by exposure to natural light; it is therefore, not dependent upon any foreign source, but inheres as a property, in a peculiarly organized animal substance or fluid, and is regulated by the same laws which govern all the other functions of living beings.

The light of the sea is always produced by living animals, and most frequently by the presence of the medusa scintillans. When great numbers of this species approach the surface, they sometimes coalesce together, and cause that snowy or milky appearance of the sea, which is so alarming to navigators. These animals, when congregated on the surface of the water, can produce a flash of light, somewhat like an electric corruscation. When the luminous medusæ are very numerous, as frequently happens in confined bays, they form a considerable portion of the mass of the sea, at which times they render the water heavier, and more nauseous to the taste; it is therefore adviseable to always strain sea water before it is drunk.

The luminous property does not appear to have any connection with the œconomy of the animals that possess it, except in the flying insects, which by that means discover each other at night, for the purpose of sexual congress.

